# ﻿The first epiphytic species of *Valeriana* in the world: *Valerianarudychazaroi* (Caprifoliaceae)

**DOI:** 10.3897/phytokeys.236.110905

**Published:** 2023-12-18

**Authors:** Antonio Francisco-Gutiérrez, Miguel Cházaro-Basáñez, Rodrigo Carral-Domínguez

**Affiliations:** 1 Facultad de Biología, Universidad Veracruzana, Circuito Universitario Gonzalo Aguirre Beltrán s.n., Zona Universitaria 91000, Xalapa, Veracruz, Mexico Universidad Veracruzana Xalapa Mexico; 2 Dirección de Recursos Naturales, Secretaría de Medio Ambiente del Estado de Veracruz, Anastacio Bustamante esq. Manlio Fabio Altamirano s.n., Centro 91000, Xalapa, Veracruz, Mexico Dirección de Recursos Naturales, Secretaría de Medio Ambiente del Estado de Veracruz Xalapa Mexico

**Keywords:** Cloud forest, Dipsacales, endemic species, epiphytic species, Mexico

## Abstract

The currently known species of *Valeriana* are herbs, shrubs, small trees and vines. After 20 years without new species of *Valeriana* in Mexico, here is described and illustrated the first epiphytic species in the genus. The species was found growing on *Quercusglabrescens* trees of the cloud forests from central Veracruz in eastern Mexico. It is known and described from very few specimens in the type locality. The most morphologically similar Mexican species are the vines *V.naidae* and *V.subincisa*, it was compared. Conservation assessment classifies this species under the Critically Endangered CR B1+B2ab(ii,v) category of the IUCN Red List Criteria.

## ﻿Introduction

*Valeriana* L. (Dipsacales, Caprifoliaceae, Valerianoideae) is a genus of ca. 270 species distributed in southern Africa, the Americas and Eurasia. Its species are annual or perennial rhizomatous herbs, often semi-rosulate or rosulate, shrubs or subshrubs, small trees or lianas (Weberling and Bittrich 2016; Rabuske-Silva et al. 2020). Barrie (2003) states North American valerians are rhizomatous or tap-rooted perennial herbs, while South American species are herbs, shrubs and vines. The highest species richness, centres of origin and centres of diversification of *Valeriana* are in the southern Andes (Bell and Donoghue 2005; Bell et al. 2012; Luebert and Weigend 2014).

Recent phylogenetic and phylogenomic analyses classify *Valeriana* into the Valerianoideae clade of Caprifoliaceae and suggest the polyphyly of the genus (Bell and Donoghue 2005; Hidalgo et al. 2010; Lee et al. 2021; Wang et al. 2021). Further studies are needed to reveal the internal relationships in *Valeriana*. As a result of this, the morphological classification is followed to maintain a coherent group, *Valeriana* s.l., to avoid multiple generic segregations until most species be sequenced, as proposed by Christenhusz et al. (2018).

The most significant contribution to the knowledge of the Mexican *Valeriana* species was performed by Barrie (2003), where seven species and one variety were described, including a key for 39 species. In the same issue of the former publication, Rzedowski and Calderón de Rzedowski (2003a) published another two new endemic species from Mexico. Later, the taxonomic treatment for *Valeriana* species of the Bajío Region in central-western Mexico was published by the same authors (Rzedowski and Calderón de Rzedowski 2003b). Since that date, no new species for Mexico have been described. The only update to the known diversity of the genus in Mexico was the addition of *V.insignis* (Suksd.) Christenh. & Byng, with distribution from California to Washington, Arizona, US and Baja California, Mexico.

In contrast, in the last five years, many *Valeriana* species from South America have been described: *V.plateadensis* Á.J.Pérez, C.H.Perss. & J.N.Zapata, *V.yacuriensis* Sklenář & B.Eriksen, *V.xenophylloides* Sklenář & B.Eriksen (Persson et al. 2023), *V.praecipitis* A.E.Villarroel & Menegoz (Villarroel et al. 2022), *V.caparaoensis* Rabuske, Sobral & Iganci (Rabuske-Silva et al. 2020), *V.nahuelbutae* Penneck. (Penneckamp 2020), *V.sobraliana* Rabuske & Iganci (Rabuske-Silva and Vieira-Iganci 2019), *V.iganciana* Rabuske & J.Külkamp (Rabuske-Silva and Külkamp 2018) and *V.vilcabambensis* Sylvester & Barrie (Sylvester et al. 2018). Recently, the medicinal species *V.officinalis* gained great importance for being an important alternative in the treatment of anxiety disorders, insomnia and stress caused during the Covid-19 pandemic around the world (Frost et al. 2021; Pessolato et al. 2021; Bertuccioli et al. 2022).

Current botanical research has discovered striking and remarkable new species with evolutionary innovations for the genus to which they belong, like *Pinangasubterranea* Randi & W.J.Baker, the first known palm species flowering and fruiting underground (Randi et al. 2023; Kuhnhäuser et al. 2023) or *V.rupicola* Pansarin & E.L.F.Menezes, the first Neotropical rupicolous species of *Vanilla* (Pansarin and Fernandes-Menezes 2023). This paper describes the first epiphytic member of the genus *Valeriana* (Caprifoliaceae) in the world found in Mexico. The aims of this study are: 1) to describe and illustrate a new species of *Valeriana*; 2) to compare the new taxon with the known species from Mexico and 3) to evaluate the conservation status of the new species.

## ﻿Materials and methods

This species was discovered in 2012 during botanical expeditions of Dr Miguel Cházaro-Basáñez (1949–2023), Dr Pablo Carrillo-Reyes and MSc David Jimeno-Sevilla in the Municipality of Tlacolulan, central Veracruz, Mexico. Miguel Cházaro determined this species as a new taxon and brought preserved specimens to Dr Jerzy Rzedowski (1926–2023) to confirm the status, obtaining the confirmation of this being a new species. A new collection was made by Miguel Cházaro-Basáñez and Rodrigo Carral-Domínguez in September 2020 to obtain specimens, geographic data and photographic evidence of the habit of this species.

### ﻿Taxonomic determination

A literature revision was carried out to identify the taxon. The species was determined following the dichotomic key of the Mexican species of *Valeriana*, published by Barrie (2003) and compared with the species described by Rzedowski and Calderón de Rzedowski (2003a). Since that date, novelties and nomenclatural changes of species distributed in Mexico have been looked for. Only the nomenclatural change of *V.insignis* (Suksd.) Christenh. & Byng, based on the basionym *Aligerainsignis* Suksd. (Christenhusz et al. 2018), was found. The synonym of the latter, Plectritisciliosavar.insignis (Suksd.) Dempster was treated by Moore (2012) in the Jepson eFlora of California as distributed in Baja California, Mexico. Morphological comparisons of similar species were made with the descriptions included in Barrie (2003) and Rzedowski and Calderón de Rzedowski (2003b).

### ﻿Conservation assessment

Geographical coordinates were obtained in the field with a Garmin eTrex 10 GPS. Data were used for calculating geographic ranges of Area of Occupancy (AOO) and Extent of Occurrence (EOO) in the Geospatial Conservation Assessment Tool (GeoCAT, Bachman et al. (2011)), available at http://geocat.kew.org. Both estimates are required by the guidelines of the IUCN (IUCN Standards and Petitions Committee 2022) for conservation assessments. Scientific literature about threats in the distribution area was searched to select the risk category accurately.

### ﻿English language revision

The artificial intelligence tool Grammarly Premium was used to corroborate the grammar and syntaxis of the manuscript.

## ﻿Taxonomic treatment

### ﻿ Valeriana rudychazaroi

Taxon classificationPlantaeDipsacalesCaprifoliaceae

Cházaro, Franc.Gut. & J.R.Carral sp. nov.

A3C034C5-336B-5BB0-9774-74000045CA7D

urn:lsid:ipni.org:names:77332878-1

#### Diagnosis.

*Valerianarudychazaroi* can be distinguished from all the known species of the genus by its epiphytic habit on trees of *Quercusglabrescens* Benth. (vs. herbs, shrubs, subshrubs, small trees or climbing vines in the rest of the genus). It is morphologically similar to *V.naidae* Barrie and *V.subincisa* Benth., from which it differs by having thinner stems (0.25–0.6 cm vs. up to 2 cm in diameter in both species), leaves elongately spatulate (vs. ovate to elliptic or narrowly ovate to elliptic, respectively), inflorescence corymboid (vs. paniculoid in both species), inserted stamens in flowers (vs. exserted in both species), different shape of fruits (ovate vs. oblong to lanceolate in both species) and longer fruits (3–5 mm vs. 2–3 mm in both species).

#### Type.

Mexico. Veracruz: Municipio Tlacolulan, Cerro de la Magdalena, 19°43'21"N, 96°59'09"W, 2900–2950 m elev., 20 September 2020, fl., fr., *R. Carral-Domínguez & M. Cházaro-Basáñez 766* (holotype: XAL!).

#### Description.

**Figure 1.**
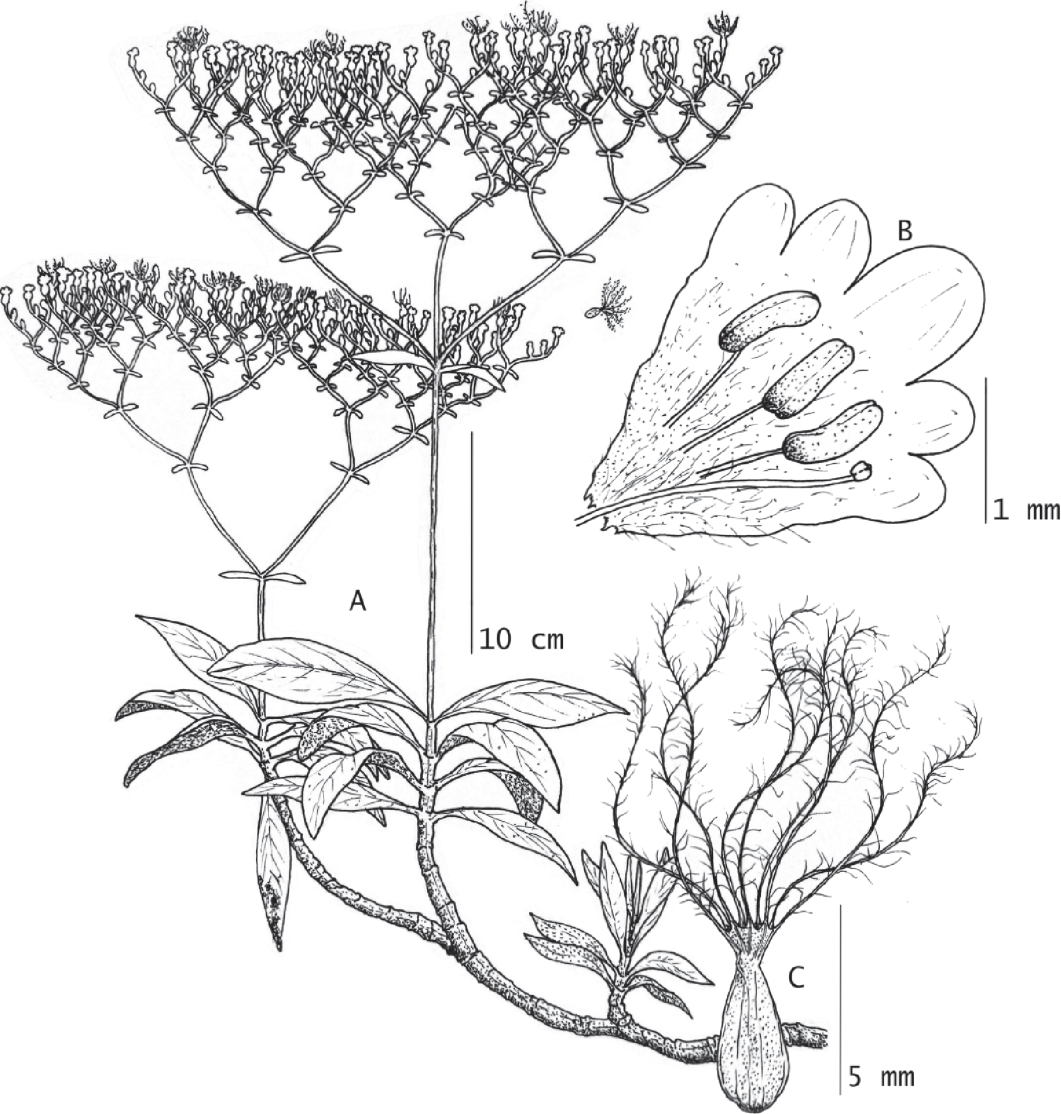
*Valerianarudychazaroi* Cházaro, Franc.Gut. & J.R.Carral. **A** habit of flowering branch **B** staminate flower **C** fruit. Drawn from the holotype *Carral-Domínguez & Cházaro-Basáñez 766* (XAL). Illustration by Gerardo Andrade–Quintos.

**Figure 2.**
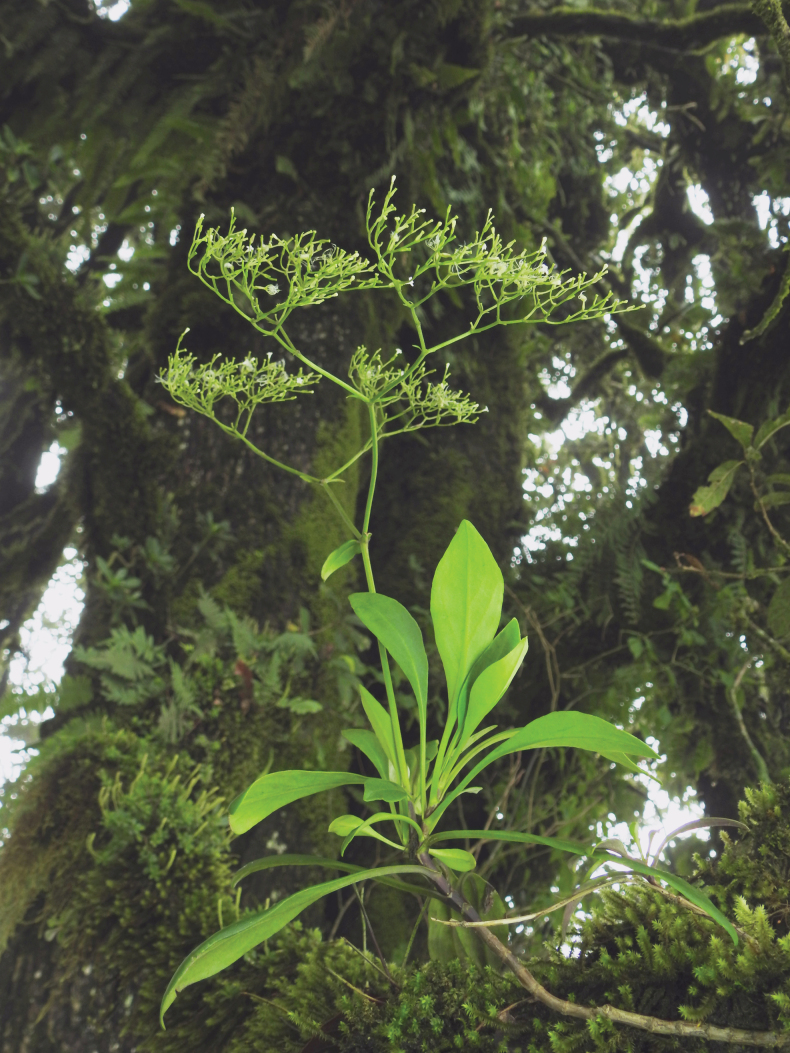
*Valerianarudychazaroi* on *Quercusglabrescens* trees in the field. Photograph taken by Rodrigo Carral-Domínguez.

**Figure 3.**
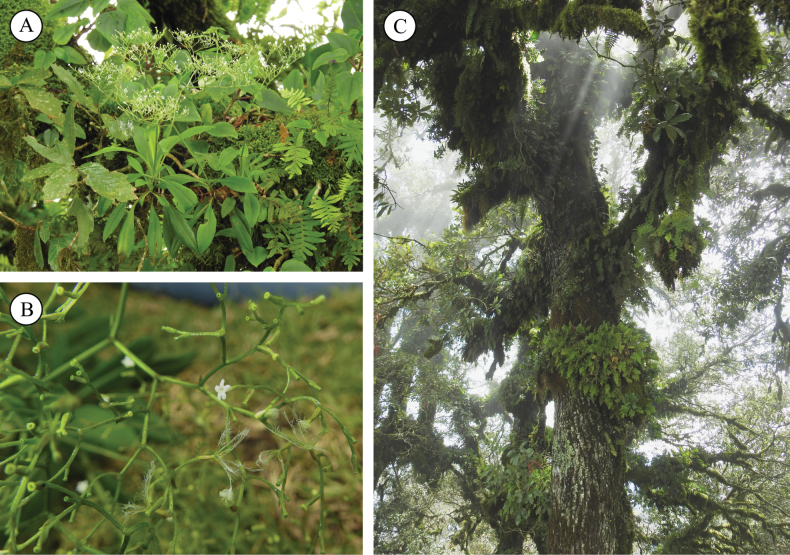
*Valerianarudychazaroi* in the field. **A** habitat at an altitude of 3–6 m on *Quercusglabrescens* trees **B** detail of inflorescence with flowers and fruit **C** hosts in cloud forests from central Veracruz, Mexico. Photographs taken by Rodrigo Carral-Domínguez.

***Perennial gynodioecious epiphyte***, growing on branches of *Quercusglabrescens*, 45–80 cm tall. ***Roots*** fibrous. ***Stems*** terete, decumbent, 20–45 × 0.25–0.6 cm, branched in the basal portion, glabrous until the insertion of the central axis of the inflorescence, where is shortly pubescent with trichomes simple, trichomes up to 0.5 mm long. ***Leaves*** cauline and clasping, simple, opposite and decussate, persistent near the inflorescence, deciduous in late phenophases, elongately spatulate, 5.7–10.8 × 0.6–2.1 cm, apex obtuse, base largely decurrent 1.5–3.5 cm long, slightly canaliculate, margin entire, one main nerve, slightly conspicuous on adaxial surface, prominent on abaxial surface, glabrous on both surfaces and margins. ***Inflorescence*** terminal, corymboid, dichotomously divided, each terminal corymb scorpioid without rotation, being less developed one of the lateral sides, 17–24 × 13–25.5 cm from the first division to the top and considering the lateral extremes of the inflorescence, main axis 10.1–21 × 0.11–0.25 cm measured from the base until the first bifurcation. Secondary axes 2, 2.9–6.0 × 0.05–0.2 cm, tertiary axes 4, 0.33–034 × 0.1 cm, decreasing dimensions as dichotomies increase, 31–85 flowers and less than five mature fruits per terminal corymb. ***Bracts*** narrowly lanceolate to lanceolate, 2.0–2.9 × 0.45–0.8 cm, base cuneate, apex acute, margin entire, glabrous, one main nerve. ***Bractlets*** of first division linear, longer than the fruits, 0.7–0.8 × 1–1.5 mm, base narrowly clasping, apex acute, margin entire, glabrous. Bractlets of corymbs linear, equal or shorter than the fruits, 1.5–5 × 0.5–0.8 mm. ***Staminate flowers*** white, 1.5 × 0.5 mm, calyx reduced, glabrous, corolla infundibuliform, tube 2–2.7 × 2 mm (opened), 5-lobed, corolla lobes elliptic to widely triangular, 0.5–0.8 × 0.4–0.5 mm, internally and externally glabrous, stamens 3, 1 mm long, adnate to the corolla in the middle of the length, anthers globose, 0.5–0.8 mm long, bithecal, glabrous; pistilodium 1.6 mm long, included, glabrous. ***Pistillate flowers*** white, 2 × 0.7 mm, calyx reduced, glabrous, corolla infundibuliform, tube 1.0–2.2 mm long, 5-lobed, corolla lobes orbicular, 1 mm in diameter, main style 2.7–5 mm long, exserted, glabrous; secondary styles reminiscent, inserted near 1/3 corolla length. ***Fruit*** a cypsela, ovate, 12 plumose limbs derived from calyx, 3–5 × 1–1.3 mm, with 3 veins on the abaxial side 1 on the adaxial side and 2 along the margins, glabrous on all surfaces (Figs 1–3).

#### Phenology.

Flowering and fruiting recorded only in September.

#### Distribution and habitat.

**Figure 4.**
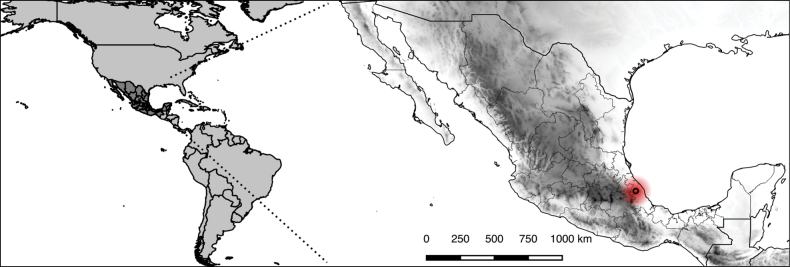
Distribution map of the epiphytic species *Valerianarudychazaroi* in Mexico.

*Valerianarudychazaroi* is only known from the type locality in cloud forests from central Veracruz in eastern Mexico (Fig. 4). There are no specimens deposited in major Mexican herbaria because of the rarity of the individuals and the difficult access to the branches of the hosts. The first collections of the species (previous to 2017) have been lost due to the death of Miguel Cházaro. During one decade of botanical explorations in the Cerro de la Magdalena Mountain and adjacent regions for floristic inventories and species descriptions (Lascurain–Rangel et al. 2017; Francisco-Gutiérrez et al. 2023a), very few specimens have been found and collected in the same locality of the type, some of them preserved as sterile material. The new species grows on very tall *Quercusglabrescens* (Fagaceae) trees, at altitudes of 3–6 m. It is distributed in the remnants of very humid pine-oak forests at elevations from 2,900 to 2,950 m. This species inhabits a zone of cloud forests on cliffs with strong winds rising from the Sierra de Chiconquiaco, Veracruz. The Sierra de Chiconquiaco is a biodiverse basin, home to 3016 species, the type localities of 72 species and 36 endemic species (Castillo–Campos et al. 2005; Lascurain-Rangel et al. 2017). The species is only known from the Volcán de la Magdalena Mountain in Tlacolulan, State of Veracruz, in eastern Mexico. From this mountain, the narrowly endemic species *Salviachazaroana* B.L.Turner (Lamiaceae), *Lobeliabiflora* Rzed. (Campanulaceae) and *Castilleja eggeri* Franc.Gut. & Cházaro were described. Species sharing the habitat are *Beschorneriayuccoides* K.Koch (Asparagaceae, Agavoideae), *Ageratinachazaroana* B.L.Turner (Asteraceae, Eupatorieae) and the epiphytic *Nelsonianthustapianus* (B.L.Turner) C.Jeffrey (Asteraceae, Senecioneae).

#### Etymology.

**Figure 5.**
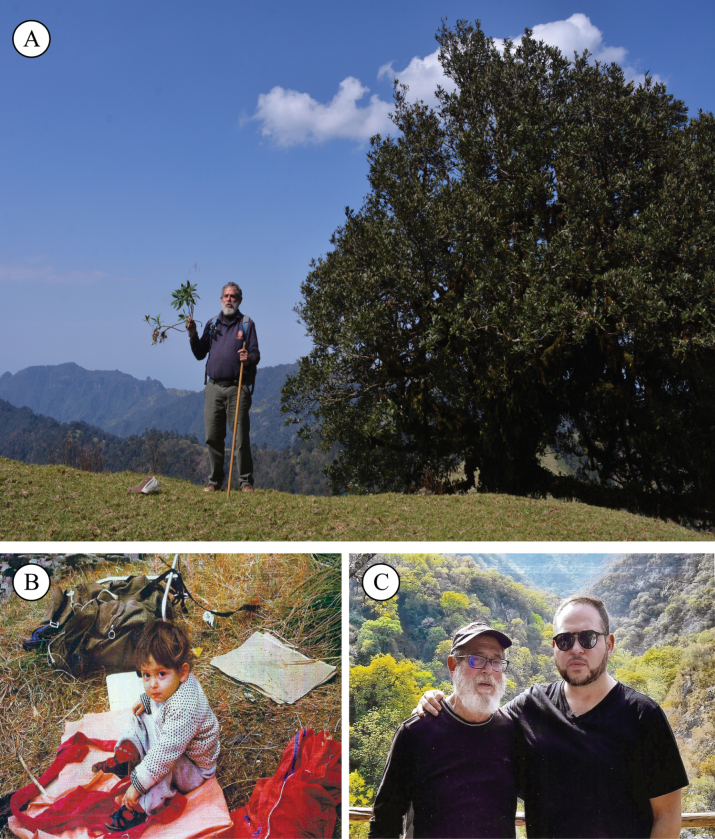
Author and eponymy of the new species **A** Miguel Cházaro showing a specimen of *Valerianarudychazaroi* next to its host, a *Quercusglabrescens* tree in Tlacolulan, Veracruz, Mexico (Author: Rodrigo Carral-Domínguez) **B** Rudy Miguel Cházaro-Hernández, son of Miguel Cházaro, during a botanical expedition at Barranca de Ramírez, 1981 **C** Miguel Cházaro and his son, to whom he dedicates the name of this new species (Author of **B** and **C**: Patricia Hernández-Romero).

Miguel Cházaro dedicates the name of the species to Rudy Miguel Cházaro-Hernández, his beloved son, who, since an early age, has accompanied him on numerous botanical trips (Fig. 5). This is the second of a series of new species that Miguel Cházaro wished to dedicate to his children before he died on 4 April 2023. First, the species *Eugeniasarahchazaroi* Cházaro, Franc.Gut. & J.R.Carral was dedicated to his daughter, Sarah M. Cházaro-Hernández (Francisco-Gutiérrez et al. 2023b). A sketch of the life of Miguel Cházaro can be consulted in his obituary (Francisco-Gutiérrez and Vázquez-García 2023).

#### Conservation status.

The new species has an Area of Occupancy (AOO) of 4 km^2^ and Extent of Occurrence (EOO) of 0 km^2^. A worrying situation for the conservation of this remarkable species is the overexploitation of oak trees that have been felled for charcoal production since the 1930’s decade (Flores 1938). Besides, the cloud forest is the most endangered ecosystem in Mexico, with projections of high vulnerability in the face of climate change scenarios (Ponce-Reyes et al. 2012). That is why, given the reduced values of AOO < 100 km^2^, EOO < 10 km^2^, number of locations = 1 and observed decline in quality of habitat, *Valerianarudychazaroi* is classified under the Critically Endangered CR B1+B2ab(ii,v) category.

#### Discussion.

*Valerianarudychazaroi* is the first recorded epiphytic species in the genus. Previous works on worldwide *Valeriana* species reported habits of small trees, herbs, subshrubs, shrubs or lianas (Weberling and Bittrich 2016). Barrie (2003) reported five species of suffruticose or herbaceous vines in the country, while the checklist of Mexican lianas reported one, *V.subincisa* (Ibarra-Manríquez et al. 2015). Unlike the *Valeriana* species that are lianas, this species has short stems that are rooted on the branches of *Q.glabrescens* trees, flowering and fruiting without contact with the ground. Further studies on seed dispersal and the biology of the species are needed.

Barrie (2003) stated that there are seven species of *Valeriana* vines in the Americas, four endemic to the northern Andes, one endemic to Panama and Costa Rica and two in Mexico: *V.naidae* and *V.subincisa*. Following the dichotomic key provided in Barrie (2003) and considering the habit of this new species, it is closest related to this group, compared to the herbaceous species. Due to the absence of twining stems, the key for species showed the new species to be most similar to *V.naidae* and *V.subincisa*, to which it was compared. A detailed comparison is provided in Table 1. The contrasting differences in morphological characters and ecological features allow us to separate *V.rudychazaroi* from other species that overlap its distribution in western Veracruz in the Cofre de Perote Volcano (Barrie 2003).

**Table 1.** Comparison of morphological characters and phenology amongst the two climbing vines and the new epiphytic species of *Valeriana* from Mexico.

Character	*V.rudychazaroi*	*V.naidae*	*V.subincisa*
	Gynodioecious	Dioecious	Gynodioecious
Habit	Epiphytic herbs	Suffrutescent herbs or climbing vines	Herbs or climbing vines
Stems length	Up to 0.8 m	Up to 15 m	1–2 m (up to 10 m when climbing)
Stems diameter	0.25–0.6 cm	Up to 2 cm	Up to 2 cm
Leaves shape	Elongately spatulate	Ovate to elliptic	Narrowly ovate to elliptic
Leaves size	5.7–10.8 × 0.6–2.1 cm	1.5–8.7 × 0.8–4.1 cm	2–8 × 0.8–4 cm
Leaves apex	Obtuse	Acute	Acute
Leaves base	Largely decurrent	Connate	Cuneate to truncate
Inflorescence type	Corymboid mostly dichotomous with terminal branchlets scorpioid.	Paniculoid with terminal branchlets scorpioid.	Panicles mostly dichotomous with terminal branchlets scorpioid.
Stamens position	Inserted	Weakly to strongly exserted	Exserted
Fruit shape	Ovate	Oblong to lanceolate	Oblong to lanceolate
Fruit length	3–5 mm	2.2–2.7 mm	2–3 mm
Phenology	Flowering and fruiting only known from September	Flowering and fruiting October-May (Flowering March-June, fruiting May-July in Nevado de Colima.	Flowering November-July
Habitat and distribution	*Quercusglabrescens* cloud forests from central Veracruz	Fir forests, cloud forests from the Trans-Mexican Volcanic Belt	*Quercus* and *Pinus* humid forests, cloud forests from Tamaulipas and Nuevo León south to Veracruz (growing along the ground), also in Chiapas and Guatemala (generally scandent).
Source	This study	Barrie (2003)	Rzedowski and Calderón de Rzedowski (2003b), Barrie, pers. comm.

## Supplementary Material

XML Treatment for Valeriana rudychazaroi

## ﻿Additional information

### Author ORCIDs

Antonio Francisco-Gutiérrez https://orcid.org/0000-0003-2013-9811

Miguel Cházaro-Basáñez https://orcid.org/0000-0003-3709-2394

Rodrigo Carral-Domínguez https://orcid.org/0000-0002-3347-5753

### Data availability

All of the data that support the findings of this study are available in the main text.
